# Quantification of neuroinflammation in spinal cord and neuroforamina of patients with painful cervical radiculopathy using [^11^C]DPA713 PET/CT

**DOI:** 10.3389/fnume.2025.1569991

**Published:** 2025-08-21

**Authors:** Ivo J. Lutke Schipholt, Gwendolyne G. M. Scholten-Peeters, Meghan A. Koop, Michel W. Coppieters, Ronald Boellaard, Elsmarieke van de Giessen, Bastiaan C. ter Meulen, Marieke Coenen, Carmen Vleggeert-Lankamp, Paul R. Depaauw, Bart N. M. van Berckel, Adriaan A. Lammerstma, Maqsood Yaqub

**Affiliations:** ^1^ Department of Human Movement Sciences, Faculty of Behavioural and Movement Sciences, Amsterdam Movement Sciences - Program Musculoskeletal Health, Vrije Universiteit Amsterdam, Amsterdam, Netherlands; ^2^ Laboratory Medical Immunology, Department of Clinical Chemistry, Amsterdam University Medical Centre, Location VUmc, Amsterdam, Netherlands; ^3^ School of Health Sciences and Social Work, Griffith University, Brisbane/Gold Coast, QLD, Australia; ^4^ Department of Radiology and Nuclear Medicine, Vrije Universiteit Amsterdam, Amsterdam University Medical Centre, Location VUmc, Amsterdam, Netherlands; ^5^ Department of Radiology & Nuclear Medicine, Amsterdam UMC, Location University of Amsterdam, Amsterdam, Netherlands; ^6^ Department of Neurology, OLVG Amsterdam, Amsterdam, Netherlands; ^7^ Department of Epidemiology and Biostatistics Amsterdam Movement Sciences Research Institute, Amsterdam UMC, Vrije Universiteit Amsterdam, Amsterdam, Netherlands; ^8^ Department of Human Genetics, Radboud Institute for Health Sciences, Radboud University Medical Center, Nijmegen, Netherlands; ^9^ Department of Neurosurgery, Leiden University Medical Center, Leiden, Netherlands; ^10^ Department of Neurosurgery, Elisabeth-TweeSteden Ziekenhuis, Tilburg, Netherlands; ^11^ Department of Nuclear Medicine and Molecular Imaging, University of Groningen, University Medical Center Groningen, Groningen, Netherlands

**Keywords:** neuroinflammation, neuropathic pain, PET imaging, cervical radiculopathy, immunoactivation, PET quantification data collection

## Abstract

**Background:**

Animal models of nerve compression have revealed neuroinflammation not only at the entrapment site, but also remotely at the spinal cord. However, there is limited information on the presence of neuroinflammation in human compression neuropathies. The objectives of this study were to: (1) assess which tracer kinetic model most optimally quantified [^11^C]DPA713 uptake in the spinal cord and neuroforamina in patients with painful cervical radiculopathy, (2) evaluate the performance of linearized methods (e.g., Logan) and simplified (e.g., standardized uptake value - SUV) methods, and (3) assess the test-retest reliability of these methods. Microglia activation associated with neuroinflammation was quantified using positron emission tomography (PET) with the radiotracer [^11^C]DPA713, targeting the 18 kDa translocator protein (TSPO). The Akaike information criterion, visual inspection of the fits and number of outliers were used to select the optimal kinetic model. As unaffected tissue, the spinal cord and neuroforamina three cervical levels above the affected target tissue was used.

**Results:**

The single tissue (1T2k) compartment model was the preferred model to describe [^11^C]DPA713 kinetics at the spinal cord and neuroforamina. Higher levels of 1T2k *V*_T_ were observed in the affected neuroforamina and spinal cord compared with corresponding unaffected tissues. Logan *V*_T_ (≥0.73) showed high correlation with 1T2k *V*_T_ at both locations. Of the simplified methods, neuroforamina and spinal cord SUV normalized for the metabolite corrected plasma (TBR-PP) exhibited high correlations with 1T2k *V*_T_ (r ≥ 0.84). Test-retest reliability varied between fair to excellent.

**Conclusions:**

These results indicate that a 1T2k model with metabolite corrected image derived input function can be used to describe the kinetics of [^11^C]DPA713 in the spinal cord and neuroforamina in humans. 1T2k *V*_T_ or Logan *V*_T_ can be used as binding metric, while TBR-PP is the recommended choice among simplified models.

## Introduction

Following peripheral nerve compression, neuroimmune activation develops in the dorsal root ganglia (DRG) and spinal cord (1). These neuroimmune responses are thought to play an important role in the pathogenesis of painful compression neuropathies, including painful lumbar and cervical radiculopathy (2–4). The further development and refinement of clinical methods to evaluate neuroimmune activation *in vivo* in the DRG and spinal cord would advance research in painful compression neuropathies in humans (2, 5).

Microglia are considered the resident macrophages of the nervous system, responsible for phagocytosis of cellular debris, antigen presentation and a variety of other functions, such as cytokine signaling (6, 7). Activated microglia and astrocytes release inflammatory mediators such as reactive oxygen species (ROS), cytokines, and neurothrophic factors that activate or sensitize the nociceptive circuitry (6, 7). Microglia express translocator protein (TSPO), an intracellular protein formerly known as the peripheral benzodiazepine receptor, which becomes overexpressed under inflammatory conditions (8, 9). Using molecular imaging techniques, such as positron emission tomography (PET), it is possible to quantify TSPO levels *in vivo* (10).

[^11^C]DPA713 is a second generation TSPO radioligand, which shows higher specific binding and better kinetic properties than the more widely used first generation TSPO ligand (R)-[^11^C]PK11195 (11–13). Nevertheless, there are challenges specific to TSPO imaging, such as polymorphisms of the TSPO gene, cellular heterogeneity of TSPO in nervous tissue and TSPO distribution in blood and plasma (5, 14). To overcome these issues, only high-affinity and mixed-affinity binders can be assessed and an input function is necessary for kinetic modelling (5). To obtain the input function, in general, arterial cannulation is required. However, when a region devoid of specific binding exists, an image derived input function is an attractive non-invasive alternative to arterial sampling (15, 16). In most cervical radiculopathies, the lower cervical levels are affected. As a result, both ascending aorta and affected nerve roots can be in the same field-of-view using a short axial field-of-view PET/CT scanner (17).

Although the optimal tracer kinetic model for quantification of [^11^C]DPA713 uptake in the brain has been described, this still is an open question for the spinal cord and neuroforamina. In addition, information on the test-retest reliability of [^11^C]DPA713 for the spinal cord and neuroforamina has not been published yet, limiting the interpretation of research findings. Therefore, the aims of the present study were (1) to evaluate a tracer kinetic models with image derived input function to quantify [^11^C]DPA713 uptake in the spinal cord and neuroforamina in patients with painful cervical radiculopathy, (2) to use this model to assess the performance of linearized [e.g., Logan (18)] and simplified [e.g., standardized uptake value (SUV) (19)] methods, and (3) to assess test-retest reliability of the resulting outcome parameters.

## Materials and methods

This study was approved by the Medical Ethics Committee of Amsterdam University Medical Centre, location VUmc (Approval number: 2020.179) and was registered at the WHO International Clinical Trials Registry Platform (https://trialsearch.who.int; study ID: NL8060). Informed consent was obtained from all individual participants included in the study.

### Study designs

**Figure 1:**
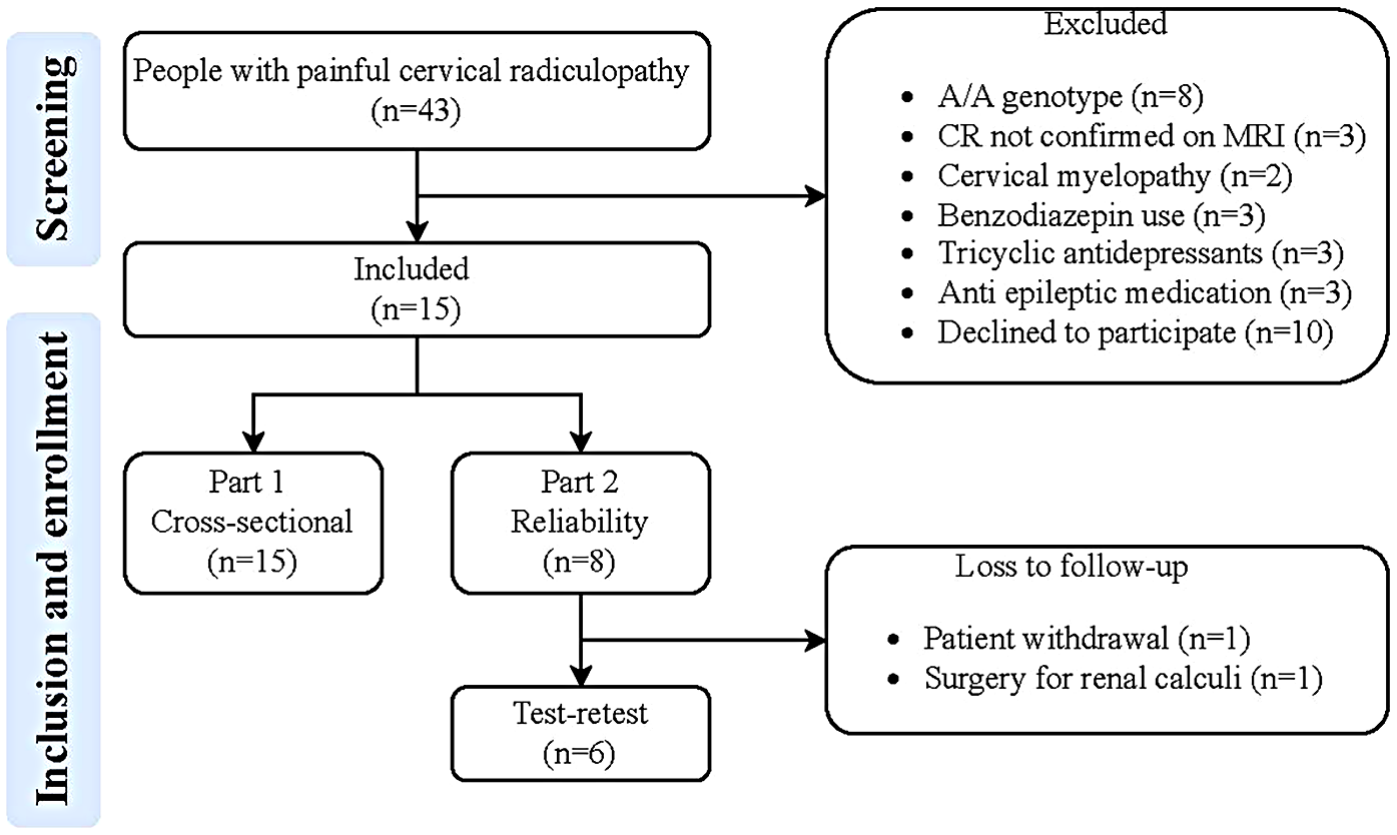
Flowchart of the study. MRI, magnetic resonance imaging; CR, painful cervical radiculopathy; G/G genotype: low-affinity binders.

The study consisted of two parts: the first part employed a cross-sectional design, while in the second part a test-retest design with a one-week follow-up was adopted. The first part of the study involved patients with a painful cervical radiculopathy to identify the optimal tracer kinetic model with an image derived input function (Figure 1) for describing uptake of [^11^C]DPA713 in the spinal cord and neuroforamina. The second part of the study focused on determining test-retest reliability of the corresponding model parameters. From the initial group of patients with painful cervical radiculopathy, a subset of six was randomly selected. These six patients were scanned on two separate occasions, with a one-week interval between measurements.

### Participants

Patients who were at least 18 years of age were eligible for inclusion if they had been diagnosed with a painful cervical radiculopathy by a medical specialist, supported by relevant Magnetic Resonance Imaging (MRI) findings. Exclusion criteria included: (1) exposure to research and medical procedure dose radioactivity with a dose above 5 mSv in the previous year, (2) use of immunosuppressive medication (e.g., corticosteroid therapy, opioid therapy, benzodiazepine use), (3) unwillingness to refrain from nonsteroidal anti-inflammatory drug (NSAID) exposure in the 2 weeks prior to scanning, (4) a cervical epidural steroid injection in the preceding 12 weeks, (5) pregnancy and/or breastfeeding, and (6) having the low affinity binding TSPO G/G polymorphism. The genotype must be performed at a prior visit, before enrolling into the protocol.

### Clinical assessment

Demographic and clinical data were obtained after inclusion. Clinical characteristics were assessed through clinical (neurological) examination and questionnaires [neck disability index (NDI) and painDETECT]. Details are provided in Table 1.

**Table 1:** Description of physical examination and questionnaires used.

Clinical assessment	Physical examination and questionnaires used	Description
Pain intensity	Visual analogue scale (VAS)	The VAS for pain is a commonly used assessment tool in healthcare to measure a patient's pain intensity. It consists of a straight line, typically 10 centimetres in length, with “no pain” at one end and “worst pain imaginable” at the other end. Patients are asked to mark the point on the line that corresponds with the intensity of their pain, providing a subjective but quantifiable measure of their pain intensity.
Myotome	n/a	C4: shoulder elevation; C5: shoulder abduction; C6: flexion elbow; C7: extension elbow; C8: wrist extension; T1: adduction thumb. Muscle strength was scored using the Medical Research Council (MRC) scale for muscle strength. It was recorded if a patient had an MRC score less than 5 (5 = normal).
Reflexes	n/a	Biceps brachii, triceps brachii reflexes. It was recorded if a patient had a hyporeflexia.
Gnostic and vital sensibility	n/a	The gnostic sensibility was assessed by gently brushing the skin area, while vital sensibility was evaluated with a sharp skin roller. Any loss of sensation was documented.
Upper-limb neurodynamic testing	ULNT1	The shoulder was depressed, the arm was abducted to 110°, and the elbow was flexed at 90°. Then, the shoulder was externally rotated to 90°, and the patient's wrist was extended. Slowly, the fingers extended, followed by the extension of the elbow. A positive test was noted when the patient experienced arm pain, which was aggravated by performing cervical lateral flexion in the opposite direction (50).
Neck disability	Neck disability questionnaire (NDI)	The NDI consists of ten items: pain intensity, personal care, lifting, reading, headaches, concentration, work, driving, sleeping, and recreation. Item scores range from 0 (no pain or limitation) to 5 (as much pain as possible or maximal limitation). The total NDI score ranges from 0 to 50 points. Higher scores indicate greater disability. The Dutch version of the NDI is a valid and responsive measure of disability (51).
Likelihood of neuropathic pain	painDETECT (PD-Q)	The PD-Q is a simple tool to predict the likelihood of a neuropathic pain component being present in persistent pain patients (52). Persistent pain was categorized into two-mechanism based groups: nociceptive and neuropathic using the PD-Q (53). The PD-Q is a reliable screening tool with high sensitivity and specificity (52). The questionnaire consists of 7 questions regarding the graduation of pain, pain course pattern and radiating pain.

### Determination of *TSPO* rs6971 genotype

The genetic variant rs6971 in *TSPO* is known to affect the binding affinity of second generation TSPO tracers (14). Therefore, patients were classified based on their genotype: homozygous AA as high affinity binders, heterozygous AG as mixed affinity binders and homozygous GG as low affinity binders. The genetic variant rs6971 was determined in DNA extracted from blood using a Chemagic STAR DNA Saliva 4k Kit (Hamilton Robotics, Reno, NV, United States), according to the manufacturer's protocol. Genotyping for rs6971 was performed using a KASP™ by Design allelic discrimination assay (LGC Genomics, Hoddesdon, UK). Genotyping was performed in a 10 µl reaction volume, comprising 10 ng DNA, 0.5 µl KASP Mastermix (low ROX), 0.125 µl KASP SNP genotyping assay (40x) and water. Each amplification for the KASPar assay was performed by an initial denaturation at 94°C for 15 min, followed by 10 cycles of denaturation at 94°C for 20 s and annealing/extension at 61°C for 60 s including a drop of 0.6°C for each cycle. This was followed by 26 cycles of denaturation at 94°C for 10 s and annealing/extension at 55°C for 60 s, followed by four cycles of denaturation at 94°C for 20 s and annealing/extension at 57°C for 60 s. This was carried out on a 7500FAST Real-Time PCR System (Applied Biosystems, Nieuwerkerk aan den IJssel, The Netherlands). Genotypes were scored using the algorithm and software (v2.0.6) supplied by Applied Biosystems. Positive controls for each possible genotype were included as quality control for the genotyping.

### Scanning protocol

Each patient was scanned on an Ingenuity TF PET/CT scanner (Philips Medical Systems, Best, the Netherlands). To ensure consistent image quality and quantification, patients were positioned so that the affected neuroforamina and ascending aorta lay within the central 10 cm of the scanner's 18.4 cm axial field of view. Positioning was confirmed via CT scout and applied uniformly across all subjects. The scan protocol consisted of a low-dose CT scan for attenuation correction, anatomical positioning, and segmentation of anatomical regions, followed by an intravenous injection of 370 ± 22 MBq [^11^C]DPA713 at the start of a dynamic PET scan of 60 min.

Acquired list mode data were reconstructed into 19 frames (1 × 15, 3 × 5, 3 × 10, 4 × 60, 2 × 150, 2 × 60 and 4 × 600 s). Data were reconstructed using 3D RAMLA in combination with CT-based attenuation correction, providing images with a final voxel volume of 4 mm^3^ (voxel dimensions: 1.7 mm × 1.7 mm × 1.7 mm in the x, y, and z directions, respectively) and a spatial resolution of 5 mm full width at half maximum (FWHM). Reconstruction included all usual corrections, such as detector normalization, and decay, dead time, attenuation, randoms and scatter corrections.

Venous blood samples were withdrawn from an intravenous catheter placed contralaterally to the tracer infusion at 5, 10, 15, 30, 35, 40, and 59 min post-injection of [^11^C]DPA713 to estimate radiolabeled metabolite fractions, plasma parent fractions, and plasma-to-whole blood ratios. Plasma was separated by centrifugation and analysed using high-performance liquid chromatography (HPLC) with offline quantification. Given the limitations of online activity detectors—particularly at later time points when plasma activity is low due to the short half-life of [^11^C] - HPLC fractions were collected and subsequently measured using a high-sensitivity multiwell counter (Wallac Wizard, PerkinElmer). This method enables reliable detection of parent and radiometabolite fractions under conditions typical of [^11^C]DPA713 studies (13, 20).

### Image segmentation

Using the CT images, regions of interest were drawn manually on the axial slices (thickness 5 mm) within the boundaries of the affected neuroforamina (containing the roots and dorsal root ganglia), spinal cord and ascending aorta using software developed in house (ACCURATE) (21, 22). Target and unaffected tissue regions of interest (ROI) were drawn using a circle with a diameter of 2 cm (1 slice; size 1.5175 cm^3^). Unaffected tissues were drawn three levels higher than the affected cervical nerve root, as primary afferent neurons that enter the spinal cord may synapse with 2nd order neurons two levels above and below the affected level due to the tract of Lissauer. As several patients with cervical radiculopathy had bilateral CR and/or symptoms, the contralateral neuroforamina could not be used as unaffected region. Preliminary research has shown that there was no difference in the volume of distribution of neuroforamina and spinal cord unaffected tissues (i.e., three levels above) between patients with cervical radiculopathy and pain-free participants, demonstrating that these anatomical regions might serve as an reference region (submitted manuscript). Target and unaffected regions were selected by a trained researcher and approved by an expert neuroradiologist. Subsequently, these ROIs were used in the corresponding dynamic [^11^C]DPA713 images in order to extract regional time-activity curves (TACs). Image derived input function (IDIF) based metabolite corrected plasma input curves were generated following a step wise procedure: (1) The input was based on a multi-exponential curve fitted separately to the discrete whole blood and plasma samples in order to extract both whole blood and plasma input curves; (2) Additionally, plasma input curves were corrected for metabolites fractions measured in plasma based on discrete blood samples fitted to a hill-fit function in order to generate metabolite corrected plasma input curve; (3) Next, to more accurately define the peak part of the input, image derived TAC before 5 min pi was scaled to match the levels of whole blood activity at 5 min and corrected for plasma-to-whole blood ratios and metabolites fractions using the curves from the first two steps and all scaled with a constant factor as such to match activity levels of blood samples at 5 min post infusion; and (4) the input function was corrected for delay relative to each TAC separately.

### Kinetic analysis

TACs were analysed using three conventional compartmental models, all requiring a metabolite corrected arterial input function. The compartment models consisted of a single tissue compartment model (1T2k), an irreversible two tissue compartment model (2T3k), and a reversible two tissue compartment model (2T4k), all including an additional parameter for the blood volume fraction.

Model selection followed a stepwise procedure aimed at minimizing bias and variability: (1) Obvious outliers (e.g., missing input function or non-physiological TACs) were excluded, (2) data were fitted using all candidate models within parameter boundariers, and preference based on the Akaike Information Criterion (AIC) for reversible vs. irreversible models was determined, (2) visual assessment of fit quality, (3) number of outlier parameter estimates, (4) robustness to noise, and (5) correlation between estimates from different compartmental models (23). Outliers were identified based on a combination of quantitative and qualitative criteria. Specifically, a case was considered an outlier if (1) the estimated volume of distribution (*V*_T_) exceeded the group median *V*_T_ for the same anatomical region by more than 50%, or (2) the time–activity curves (TACs) showed non-physiological patterns, such as abrupt signal drops, typically associated with patient motion (see Supplementary Figure S1). The optimal fitting model was used to derive various model dependent kinetic parameters such as rate constants (*K_1_*, *k_2_, k_3_, k_4_*), the net influx rate constant (*K_i_*), and the volume of distribution (*V*_T_). *V*_T_ was considered as the primary outcome parameter to describe uptake of [^11^C]DPA713 within tissues. *V*_T_ is an index of receptor density and equals the ratio at equilibrium of the concentration of [^11^C]DPA713 in tissue to that in plasma (24). The concentration of [^11^C]DPA713 in tissue represents the sum of specific binding (receptor bound) and non-displaceable uptake (non-specifically bound and free radioligand in tissue water) (24).

### Linearized and simplified method analysis

The accuracy and precision of linearized plasma input methods [either Patlak or Logan (18, 25)] and several simplified static approaches were evaluated by comparing their outcome measures with corresponding kinetic parameters obtained with the optimal non-linear compartment methods. Simplified static approaches consisted of measures of average regional tracer uptake over an interval (20–50 and 30–60 min post-injection.) normalized to (1) patient weight and injected dose (SUV: standardized uptake value), (2) activity in arterial whole blood [target-to-whole blood ratio (TBR-WB)] and (3) activity in metabolite corrected plasma [target-to-metabolite corrected plasma ratio (TBR-PP)].

### Statistical analysis

To assess the performance of a simplified method, such as SUV, a Pearson's or Spearman's rank correlation coefficient (r) was employed depending on the distribution of the data. The interpretation of correlation scores followed these categories: r < 0.30: low/insignificant correlations, 0.30 ≥ r < 0.45: moderate correlation, 0.45 ≥ r < 0.60 substantial correlation and r ≥ 0.60: high correlation (26). To assess test-retest reliability, intraclass correlation coefficients (ICC_2.1_) were calculated employing absolute agreement, two-way mixed effects models, accompanied by 95% confidence intervals (CIs) (27). For a minimum standard of test-retest reliability, ICC_2.1_ values of at least 0.76 were deemed essential (27). The interpretation of ICC_2.1_ scores followed these categories: 0.00–0.40: poor reliability, 0.41–0.75: fair to good reliability and, 0.76–1.00: good to excellent reliability (27). In addition, measures for absolute measurement error between test and retest were calculated, including the standard error of measurement (SEM) based on ICC_2.1_, the smallest detectable difference (SDD) and the Bland-Altman Limits of Agreement (Bland Altman LoA). SEM was defined by SEM=StandardDeviation* $\sqrt{(1}-ICC2.1\;)$, SDD by $SDD=SEM\;*\;\sqrt{2}\;*\;1.96$ (28), and Bland-Altman LoA (29) by Bland−AltmanLoA=±1.96*SD+meandifferencebetweenvalues. *P*-values smaller than 0.05 were considered as statistically significant. SPSS version 28 (IBM, Armonk, NY, USA) was used for the statistical analyses.

## Results

### Participants

Fifteen patients with cervical radiculopathy were included in the study and a randomly selected subset of six patients participated in the test-retest study. The C7 radiculopathy was the most commonly affected (9 out of 15), followed by C6 radiculopathy (5 out of 15) and C5 radiculopathy (1 out of 15). In the subset of cervical radiculopathy participants who took part in the test-retest study, C6 radiculopathy was the most frequently affected (3 out of 6), followed by C7 radiculopathy (2 out of 6), and C5 radiculopathy (1 out of 6). Table 2 provides an overview of baseline participant demographics, clinical characteristics and functional profiles.

**Table 2:** Overview of the baseline and test-retest participant demographics, clinical characteristics and functional profiles.

Participant characteristics	Cervical radiculopathy (*n* = 15) Mean (SD)	Test-retest reliability (*n* = 6) Mean (SD)
Age (Y)	50 (12)	55 (12)
Duration symptoms (weeks)	49 (21)	43 (18)
Sex (% male)	53	50
TSPO genotype (% high affinity)	46%	66%
Injected dose (MBq)	370 (22)	357 (25)
BMI	25 (3.5)	25 (3.1)
Neck pain intensity (mean VAS)	47 (22)	52 (19)
Arm pain intensity (mean VAS)	54 (23)	46 (20)
Neck pain intensity (max VAS)	60 (26)	56 (17)
Arm pain intensity (max VAS)	52 (24)	59 (25)
Neck disability (NDI)	20 (9.2)	25 (8.3)
Neuropathic pain (painDETECT)	16 (6.5)	18 (7.6)
ULNT1 positive	80%	67%
Reduced reflexes	100%	100%
Muscle weakness	100%	100%
Vital sensory changes	100%	100%
Gnostic sensory changes	100%	100%

SD, standard deviation; BMI, body mass index; VAS, visual analogue scale; ULNT1, upper-limb neurodynamic testing.

### Sample data

**Figure 2:**
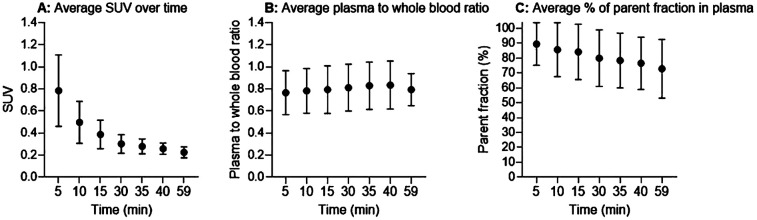
Input functions. **(A)** Normalized whole blood standardized uptake value (SUV). **(B)** Plasma-to-whole blood concentration ratio. **(C)** Average percentage of parent fractions in plasma as a function of time (minutes). Black circles are mean values and error bars represent one standard deviation.

The average normalized whole blood sample values (SUV) decreased exponentially over time with substantial individual variation (Figure 2A). The average plasma-to-whole blood ratio was approximately 0.8 during the whole scan period (Figure 2B). On average, parent tracer fractions decreased to 80% at 30 min p.i. and slowly decreased further to 72% at 60 min (Figure 2C).

### Kinetic analysis

For one patient, the kinetic analysis was not performed as the ascending aorta was outside the field of view, thereby prohibiting the creation of an image derived input function. The following boundaries for the kinetic parameters were employed: *K_1_* upper boundary (UB): 0.7, *V*_T_ UB: 40, *k_3_* UB: 0.8 and *V_b_* UB: 0.7, and all lower boundaries were set to 0. Model performance according to the Akaike information criterion (AIC) revealed that for all regions (neuroforamina and spinal cord) the reversible models (1T2k or 2T4k) were preferred over the irreversible model (2T3k) in approximately 55% of cases. The single tissue compartmental model (1T2k) was the preferred model in 11% and 32% of cases for neuroforamina and spinal cord, respectively. The 2T4k model was the preferred model for the neuroforamina in 39% of cases and in 26% for the spinal cord. In the cases where AIC indicated a preference for the 2T3k model, often were noisy due to relative low signal from small regions and AIC differences were relatively small compared with those of the reversible models. Based on visual inspection, combined for neuroforamina and spinal cord, 1T2k showed 1 and 2T4k 20 outliers in the outcome measures due to patient motion. See Supplementary Figure S1 for a visual presentation of an outlier due to patient motion for the different kinetic models. Neuroforamina 2T4k *V*_T_ was highly correlated with 1T2k *V*_T_ in both neuroforamina (r = 0.91; *p* < 0.01) (Supplementary Figure S2A) and spinal cord (r = 0.85; *p* < 0.01) (Supplementary Figure S2B). Based on the number of outliers, visual fit quality and AIC, the 1T2k model was selected as the optimal model for describing [^11^C]DPA713 kinetics in both neuroforamina and spinal cord.

### Linear and simplified analyses

**Figure 3:**
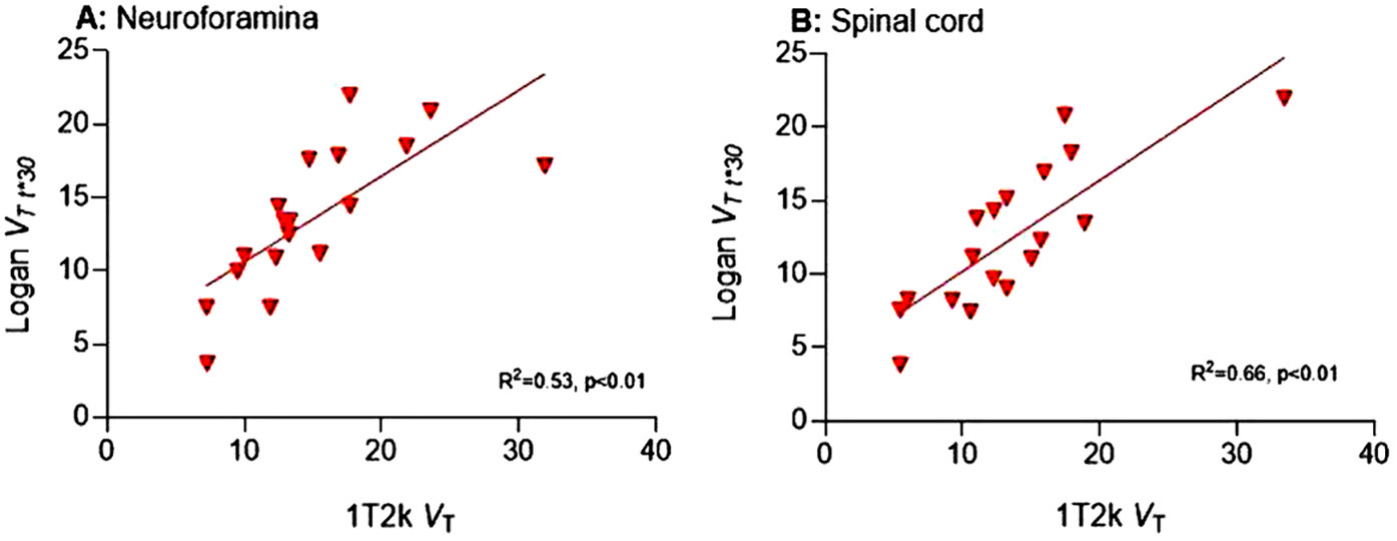
Correlation between *V*_T_ 1T2k and *V*_T_ Logan. *V*_T_, Volume of distribution; 1T2k, single-tissue compartmental model; R^2^, explained variance.

Volume of distribution *V*_T_ derived from Logan was compared with that of the preferred 1T2k model. Neuroforaminal Logan *V*_T_ (with t* = 30 min post-injection) demonstrated high correlation with 1T2k *V*_T_ (r = 0.73; *p* < 0.01) (Figure 3A). Similarly, for the spinal cord, Logan *V*_T_ (t* = 30) exhibited high correlation with 1T2k *V*_T_ (r = 0.81, *p* < 0.01) (Figure 3B).

To assess whether simplified analyses could be used, neuroforamina and spinal cord SUV and TBR for two time intervals (20–50 and 30–60 min post-injection) were compared with corresponding 1T2k *V*_T_ values. In the neuroforamina, TBR-WB (time interval 20–50: r = 0.48; *p* = 0.04; time interval 30–60: r = 0.50; *p* = 0.04) demonstrated substantial correlations with 1T2k *V*_T_. In addition, TBR-PP levels (time interval 20–50: r = 0.85; *p* < 0.01; time interval 30–60: r = 0.88; *p* < 0.01) had high correlations with 1T2k *V*_T_. In contrast, there were low correlations between SUV (time interval 20–50: r = 0.01; *p* = 0.85; time interval 30–60: r = 0.01; *p* = 0.99) and 1T2k *V*_T_.

At the spinal cord, high correlations were present between TBR-WB (time interval 20–50: r = 0.64, *p* = 0.14; time interval 30–60: r = 0.60; *p* = 0.07) with 1T2k *V*_T_ and between TBR-PP levels (time interval 20–50: r = 0.67, *p* < 0.01; time interval 30–60: r = 0.69, *p* < 0.01) and 1T2k *V*_T_. There were moderate correlations between SUV (time interval 20–50: r = 0.34; *p* = 0.14; time interval 30–60: r = 0.41; *p* = 0.07) and 1T2k *V*_T_. See Supplementary Table S3 for more details.

### Differences between target and unaffected tissues

Neuroforamina and spinal cord 1T2k *V*_T_ levels were significantly higher in the target regions compared with the unaffected tissues (Table 3). This difference was also present for Logan *V*_T_ and simplified methods (Table 3).

**Table 3:** Outcome values for the non-linear, linearized and simplified models for both target and unaffected tissues.

Outcome values	Neuroforamina target tissue mean (SD)	Neuroforamina unaffected tissue mean (SD)	Mean difference (SE)	*P*-value	Spinal cord target tissue mean (SD)	Spinal cord unaffected tissue mean (SD)	Mean difference (SE)	*P*-value
Non-linearized kinetic model								
1T2k *V*_T_	15.00 (6.09)	8.80 (3.51)	6.20 (1.62)	<0.01	13.52 (6.24)	7.41 (4.05)	6.11 (1.74)	<0.01
Linearized models								
Logan *V*_T_	12.91 (5.45)	7.65 (3.04)	5.26 (1.46)	<0.01	12.21 (4.84)	6.83 (3.04)	5.37 (1.34)	<0.01
Simplified models								
TBR-WB 30_60	7.20 (1.45)	4.67 (1.32)	2.53 (0.43)	<0.01	6.31 (1.79)	3.79 (0.97)	2.52 (0.46)	<0.01
TBR-WB 20_50	6.69 (1.43)	4.33 (1.29)	2.36 (0.42)	<0.01	5.87 (1.78)	3.51 (0.94)	2.37 (0.46)	<0.01
TBR-PP 30_60	9.30 (3.22)	5.96 (2.16)	3.34 (0.84)	<0.01	8.15 (3.20)	4.81 (1.36)	3.34 (0.79)	<0.01
TBR-PP 20_50	8.71 (2.96)	5.57 (2.07)	3.14 (0.79)	<0.01	7.64 (2.95)	4.48 (1.24)	3.16 (0.73)	<0.01
SUV 30_60	1.79 (0.29)	1.15 (0.27)	0.63 (0.09)	<0.01	1.57 (0.43)	0.95 (0.20)	0.64 (0.11)	<0.01
SUV 20_50	1.72 (0.28)	1.11 (0.28)	0.61 (0.09)	<0.01	1.52 (0.41)	0.91 (0.20)	0.61 (0.10)	<0.01

*V*_T_, Volume of distribution; 1T2k, single-tissue compartmental model; 2T3k, two-tissue irreversible compartmental model; 2T4k, two-tissue reversible compartmental model; SUV, standardized uptake value; TBR-WB, target-to-whole blood ratio; TBR-PP, target-to-metabolite corrected plasma ratio.

### Test-retest reliability

**Figure 4:**
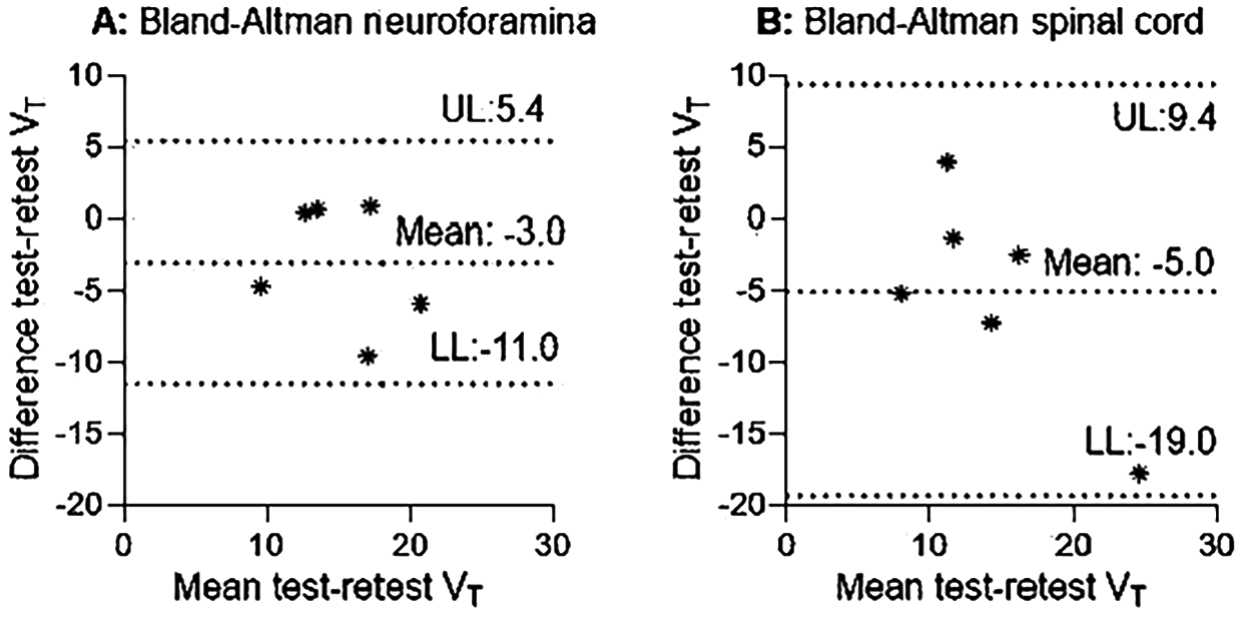
Bland-Altman of *V*_T_ test-retest data. **(A)** and **(B)** reveal the 1T2k *V*_T._

1T2k derived *V*_T_ revealed fair to good reliability for the neuroforamina and poor reliability for the spinal cord (Table 4, Figures 4A,B). In addition, 1T2k derived *V*_T_ revealed fair to good reliability for unaffected neuroforamina and poor reliability for unaffected spinal cord (Table 4). This variation in test-retest reliability might be due to variations in metabolite concentration determination, as the determined metabolite fractions substantially differed between test-retest measurements (see Supplementary Table S4). Omitting the metabolite correction (1T2k_WB) revealed good to excellent reliability for the neuroforamina and fair to good reliability for the spinal cord. Logan *V*_T_ showed good to excellent test-retest reliability with ICC_2.1_ values above 0.76 for the neuroforamina, but poor reliability for the spinal cord. Amongst the simplified measures, at the neuroforamina, SUV_30–60 revealed test-retest reliability with ICC_2.1_ values above the 0.76 threshold. At the spinal cord, TBR-PP_30–60, SUV 20–50, and SUV 30–60 showed test-retest reliability with ICC values above the 0.76 threshold. See Table 4 for more quantitative details.

**Table 4:** Test-retest reliability data per neuroinflammatory metric .

Outcome values	Test mean (SD)	Retest mean (SD)	Mean difference (SD)	r	R^2^, *p*-value	ICC_2.1_ (95%CI), *p*-value	SEM	SDD	LoA (LL, UL)
Neuroforamina									
1T2k *V*_T target tissue_	16.64 (5.02)	13.63 (3.91)	3.01 (4.31)	0.56	0.32, 0.25	0.64 (−0.55, 0.95), 0.10	2.02	5.60	−11.0, 5.4
1T2k *V*_T unaffected tissue_	9.65 (3.78)	8.12 (3.91)	−1.53 (3.62)	0.56	0.32, 0.25	0.71 (−0.69, 0.96), 0.10	5.75	5.74	−8.6, 5.6
Logan *V*_T_	15.26 (4.85)	14.21 (5.01)	1.05 (4.17)	0.64	0.41, 0.17	0.80 (−0.48, 0.97), 0.06	2.20	6.11	−9.2, 7.1
TBR_WB 20_50	6.52 (0.86)	6.42 (1.49)	−0.10 (1.67)	0.71	0.50, 0.89	0.14 (178, 0.89), 0.45	1.09	3.03	−3.4, 3.2
TBR_WB 30_60	6.83 (0.97)	7.15 (1.52)	0.32 (1.99)	0.23	0.05, 0.65	0.69 (17.37, 0.81), 0.68	1.62	4.50	−3.6, 4.2
TBR_PP 20_50	8.61 (3.30)	9.00 (3.74)	0.39 (3.56)	0.49	0.24, 0.32	0.70 (−2.23, 0.96), 0.13	1.96	5.43	−6.6, 7.4
TBR_PP 30_60	9.17 (3.98)	9.80 (4.01)	0.63 (4.25)	0.61	0.37, 0.39	0.64 (−2.85, 0.95), 0.17	2.39	6.65	−4.7, 11.0
SUV 20_50	1.81 (0.17)	1.80 (0.22)	-<0.01 (0.20)	0.49	0.24, 0.33	0.68 (−2.75, 0.96), 0.15	0.11	0.31	−0.4, 0.4
SUV 30_60	1.85 (0.22)	1.90 (0.25)	0.06 (0.20)	0.63	0.39, 0.18	0.79 (−0.53, 0.97), 0.07	0.10	0.29	−0.3, 0.5
Spinal cord									
1T2k *V*_T target tissue_	16.83 (8.87)	11.86 (3.71)	4.96 (7.32)	0.59	0.35, 0.22	0.53 (−0.85, 0.93), 0.17	4.31	11.96	−19.0, 9.4
1T2k *V*_T unaffected tissue_	6.71 (2.25)	8.32 (6.70)	1.61 (6.58)	0.22	0.05, 0.67	0.16 (−9.75, 0.90), 0.39	4.11	11.39	−11.0, 15.0
Logan *V*_T_	15.17 (6.26)	11.83 (2.62)	3.34 (6.65)	0.06	0.00, 0.91	0.07 (−3.45, 0.86), 0.47	4.28	11.88	−16.0, 9.7
TBR_WB 20_50	5.78 (1.74)	5.38 (0.69)	0.39 (1.56)	0.44	0.19, 0.39	0.49 (−4.10, 0.93), 0.25	0.86	2.40	−3.5, 2.7
TBR_WB 30_60	6.20 (2.06)	5.95 (0.76)	0.26 (1.56)	0.76	0.58, 0.08	0.69 (−1.97, 0.96), 0.13	0.78	2.18	−3.3, 2.8
TBR_PP 20_50	7.91 (4.18)	7.52 (2.55)	0.39 (3.23)	0.64	0.41, 0.18	0.75 (−1.32, 0.97), 0.09	1.68	4.74	−6.7, 5.9
TBR_PP 30_60	8.61 (4.91)	8.06 (2.56)	0.54 (3.52)	0.73	0.53, 0.10	0.77 (−0.98, 0.97), 0.08	0.79	2.18	−7.5, 6.4
SUV 20_50	1.62 (0.46)	1.57 (0.42)	0.05 (0.17)	0.94	0.88, < 0.01	0.97, (0.79, 0.99), < 0.01	0.07	0.21	−0.4, 0.3
SUV 30_60	1.69 (0.51)	1.65 (0.47)	0.04 (0.09)	0.99	0.98, < 0.01	0.99 (0.95, 0.99), < 0.01	0.04	0.13	−0.2, 0.1

*V*_T_, volume of distribution; 1T2k, single-tissue compartmental model; 2T3k, two-tissue irreversible compartmental model; 2T4k, two-tissue reversible compartmental model; SUV, standardized uptake value; TBR-WB, target-to-whole blood ratio; TBR-PP, target-to-metabolite corrected plasma ratio; ICC, intraclass coefficient; SEM, standard error of the mean; SDD, smallest detectable difference; LOA, limits of agreement; LL, lower limit; UL, upper limit; SD, standard deviation.

## Discussion

This study resulted in four main findings. First, based on AIC, visual inspection of fits and the number of outliers, the 1T2k model was the preferred model to describe [^11^C]DPA713 kinetics in neuroforamina and spinal cord of patients with painful cervical radiculopathy. Second, Logan *V*_T_ and TBR-PP showed high correlations with 1T2k derived *V*_T_ for both affected neuroforamina and spinal cord. Third, at the neuroforamina, 1T2k_WB *V*_T_, Logan *V*_T,_ and SUV_30–60 showed ICC_2.1_ values above the set threshold of 0.76. At the spinal cord, TBR-PP, and SUV showed ICC_2.1_ values above the set threshold of 0.76. Fourth, 1T2k *V*_T_, 1T2k_WB *V*_T_, Logan *V*_T_ and all simplified measures revealed elevated [^11^C]DPA713 uptake in the affected neuroforamina and spinal cord compared with unaffected tissues using a within-person design.

When comparing the present findings with previous reports, it is important to note that limited data are available on human musculoskeletal pain conditions. TSPO PET imaging has been employed as a neuroinflammatory biomarker mainly in the assessment of microglial activity in neurodegenerative diseases rather than in musculoskeletal pain conditions (30, 31). The scarcity of available data has limited the translational potential of preclinical work (32–34). In a human study comparing patients with a cervical radiculopathy and pain-free participants (*n* = 5 in each group), [^11^C]DPA713 SUV revealed no observable spinal neuroinflammation in the cervical radiculopathy group (35). A comparison with the present study is difficult, as no information was provided regarding the time interval during which SUV was measured. Another human study indicated an increase in [^11^C]PBR28 SUV in the ipsilateral neuroforamina and spinal cord segments in patients with chronic radicular pain (*n* = 19) compared with pain-free participants (*n* = 10) (2). In the present study, using a within-subject design, all binding metrics - *V*_T_, Logan *V*_T_, and simplified measures—revealed elevated tracer uptake in the spinal cord and neuroforamina compared with unaffected tissues. *V*_T_ offers advantages over simplified measures like SUV because it may accounts for blood flow, radiometabolites, and tracer clearance over time, addressing several limitations of SUV. Although simplified measures are practical and revealed between-group differences in our study, there are important scenarios where quantitative kinetic modeling is essential. Even without direct estimation of binding kinetics (*k_3_/k_4_*), 1T2k derived *V*_T_ enables a more robust interpretation of tracer behaviour than SUV alone and is particularly valuable in longitudinal designs, such as therapy monitoring or disease progression studies, where systemic physiological changes (e.g., inflammation or vascular alterations) may influence tracer delivery and confound SUV-based analyses (5, 36). Although challenges in radiometabolite assay accuracy, especially at later time points, can impact quantitative outcomes, we addressed this by employing a high-sensitivity multiwell counter validated for low-activity [^11^C]DPA713 samples (see Methods). This methodological approach helps mitigate counting-related bias and supports the robustness of the derived input function and resulting *V*_T_ estimates.

### Methodological considerations

Model selection followed a careful process. Initial exclusions were made for obvious artifacts, such as missing input function data or non-physiological TACs. The remaining datasets were analyzed using all candidate models within defined parameter boundaries, with AIC used to guide the initial decisions. Although AIC values showed a slight advantage for the 2T4k model in certain regions, such as the neuroforamina, model selection was guided by additional factors: visual fit quality, resistance to noise, prevalence of outliers, and the physiological plausibility of estimated parameters. The 2T4k model frequently produced unstable fits in smaller regions with lower signal-to-noise ratios, resulting in 20 outliers compared to only one for the 1T2k model. Many of these were associated with slight patient motion, observed in about 15% of scans. Motion artifacts were identified through visual inspection of dynamic PET/CT frames and TACs, which occasionally displayed abrupt signal drops (see Supplementary Figure S1). TACs strongly affected by motion were excluded. However, slight motion artifacts in combination with noise disproportionately affected the 2T4k model, which in response yielded exaggerated *V*_T_ values and larger standard deviations. By contrast, the more constrained 1T2k model yielded stable, physiologically plausible estimates even under these conditions. Comparing *V*_T_ estimates from both models revealed high correlations (r = 0.91 in neuroforamina, r = 0.85 in spinal cord), indicating that 1T2k can reliably estimate *V*_T_. Although AIC provided a useful initial guide, the final decision was ultimately driven by practical considerations: consistency of parameter estimates, visual fit quality, and close agreement in *V*_T_ values. Under ideal, high signal-to-noise ratio conditions, the 2T4k model might offer finer detail. However, in the practical setting of this study, the 1T2k model proved both robust and accurate. As a final consideration, the known reversibility of [^11^C]DPA713 uptake in nervous tissue supports the physiological validity of a reversible single-tissue model (37, 38).

The direct calculation of binding potential relative to the non-displaceable compartment (*BP_ND_* = *k_3_*/*k_4_*) is not possible with the 1T2k model, as *k_3_* and *k_4_* are not determined (*BP_ND_*). Alternatively, *BP_ND_* could be determined indirectly from distribution volume ratio (*DVR)* minus 1 based on a reference tissue approach. However, use of a reference region to calculate *DVR* and *BP_ND_* requires: (1) the reference region is devoid of specific/displaceable binding, (2) reference and target tissues have the same non-displaceable volume of distribution and (3) *V*_T_ can be measured reliably in both tissues (39). The unaffected tissue in this study could not be used as reference region as it does not fulfil the assumption of no specific binding. Further research will be necessary to identify and validate suitable tissues that can serve as reference tissue when calculating *DVR* and *BP_ND_* for the spinal cord and neuroforamina.

There were high correlations between Logan *V*_T_ and 1T2k *V*_T_, as well as between simplified metrics, particularly TBR-PP, and 1T2k *V*_T_. Despite the high correlations between TBR-PP metrics (SUV corrected for parent fraction in plasma) and 1T2k *V*_T_, the use of static TBR-PP entails significant limitations. Radiculopathy commonly involves edema, which can alter local blood flow within the dorsal root ganglion and, consequently, affect tracer delivery. While changes in perfusion may influence early tracer kinetics, the *V*_T_ as derived from compartmental modeling is generally considered robust to such variations, assuming sufficient scan duration and appropriate model fit. Moreover, the plasma-to-tissue influx rate constant (K_1_) provides a means to assess regional perfusion directly. In contrast, static imaging measures such as target-to-background ratio offer no information about tracer kinetics and do not account for differences in tissue perfusion, potentially introducing variability unrelated to specific tracer binding. Especially in longitudinal studies investigating treatment effects, changes in perfusion of the dorsal root ganglion may be expected, and the use of TBR metrics will need to be validated against the 1T2k model.

Single tissue compartmental model *V*_T_ only reached fair to good test-retest reliability with ICC values falling below the set cut-off point of 0.76. Uncertainty in the estimation of true metabolite fractions may contribute to variability in 1T2k *V*_T_. These inaccuracies are unlikely due to the HPLC method itself, which is validated and reliable, but rather reflect practical challenges during venous sampling, such as insufficient blood volume or prolonged sampling duration. The uncorrected metabolite model (1T2k_WB *V*_T_) revealed better test-retest reliability with an ICC of 0.88 for the affected neuroforamina and an ICC of 0.74 for the spinal cord. Logan *V*_T_ showed good to excellent reliability at the neuroforamina, but poor reliability at the spinal cord. Another reason for the suboptimal test-retest reliability could stem from the CT slice thickness of 5 mm. Given the limited axial field of view (18 cm) and the necessity for the ascending aorta to consistently remain within this field, it is plausible that the visualization of the neuroforamina was not consistent across test-retest images. This variation could have introduced inconsistency in drawing the VOI on the longitudinal plane. However, different VOI strategies (1 slice vs. more slices) were used and the adopted method showed the highest reliability. Future studies should consider the use of artificial intelligence methodologies to automate identification of the target areas of interest as an attempt to increase test-retest reliability (40).

Compared with corresponding unaffected tissues, increased [^11^C]DPA713 binding was observed at the affected neuroforamina and spinal cord, suggesting neuroinflammation. There are several factors that might cause neuroinflammation locally at the affected neuroforamina and spinal cord. Firstly, neuroinflammation might arise due to disturbed homeostasis caused by compression and/or chemical irritation of the cervical nerve root(s) and/or dorsal root ganglion. Clinical immunohistochemical analysis has shown that nucleus pulposus material initiates a chemical cascade, involving pro-inflammatory mediators, such as tumour necrosis factor (TNF), interleukin-6 (IL-6), and matrix metalloproteinases (MMPs) (41). Secondly, aberrant neuronal activity might occur, resulting in ectopic/aberrant firing of nociceptors, which induces inflammatory reactions, a process called neurogenic neuroinflammation (42–44). From a broader neuroscience perspective, this study demonstrates that PET imaging with [^11^C]DPA713 can be used to visualize neuroinflammatory processes *in-vivo* in the human spinal cord and nerve roots, regions that are otherwise difficult to access. These findings contribute to our understanding of the biological mechanisms that may underlie radiculopathy and related symptoms. Furthermore, such *in-vivo* imaging approaches provide a valuable tool to study neuroimmune interactions in the human nervous system and how these may relate to structural pathology or clinical symptoms. This may help guide future investigations into therapeutic strategies targeting neuroinflammatory pathways.

It is important to acknowledge limitations associated with the TSPO tracer, as it does not exclusively reflect microglial activation but also astrocyte involvement in neuroinflammation (45). Inter-individual genetic variations can influence TSPO binding, and therefore only individuals with mixed and high-affinity binding for [^11^C]DPA713 were included. Moreover, [^11^C]DPA713 binding to TSPO does not differentiate between anti-inflammatory and pro-inflammatory phenotypes, indicating the need for tracers capable of discriminating between these two immune responses (46). [^11^C]DPA713 has potential advantages over other tracers, such as *(R)*-[^11^C]PK11195, including increased binding affinity, and improved signal-to-noise ratio(5). To reduce patient burden during PET/CT scanning, image-derived input function was used in combination with venous sampling rather than arterial sampling. It is important to consider that image-derived input function methods are highly tracer-specific and influenced by tracer kinetics and radio metabolites (15). A limitation of this study is the lack of direct validation of the image-derived input function against arterial sampling for [^11^C]DPA713 on this scanner. However, using large blood pools, high correlations have been shown between the input function derived from arterial cannulation and that derived from the ascending aorta (ICC>0.98) (47). Partial volume effects might pose a significant problem, but they were minimal in this study as shown in the scaling factor. The use of venous blood to determine the metabolite fraction has the advantage of reducing the patient burden compared with arterial metabolite fraction determination. However, while for one tracer arterial and venous metabolite fractions may be similar, for other there may be significant differences (48). For [^11^C]DPA713, the use of venous samples to determine metabolite fractions might introduce errors when creating the image derived input function corrected for labelled metabolites. For [^11^C]DPA713, arterial and venous metabolite analyses should be compared in a future study.

**Figure 5:**
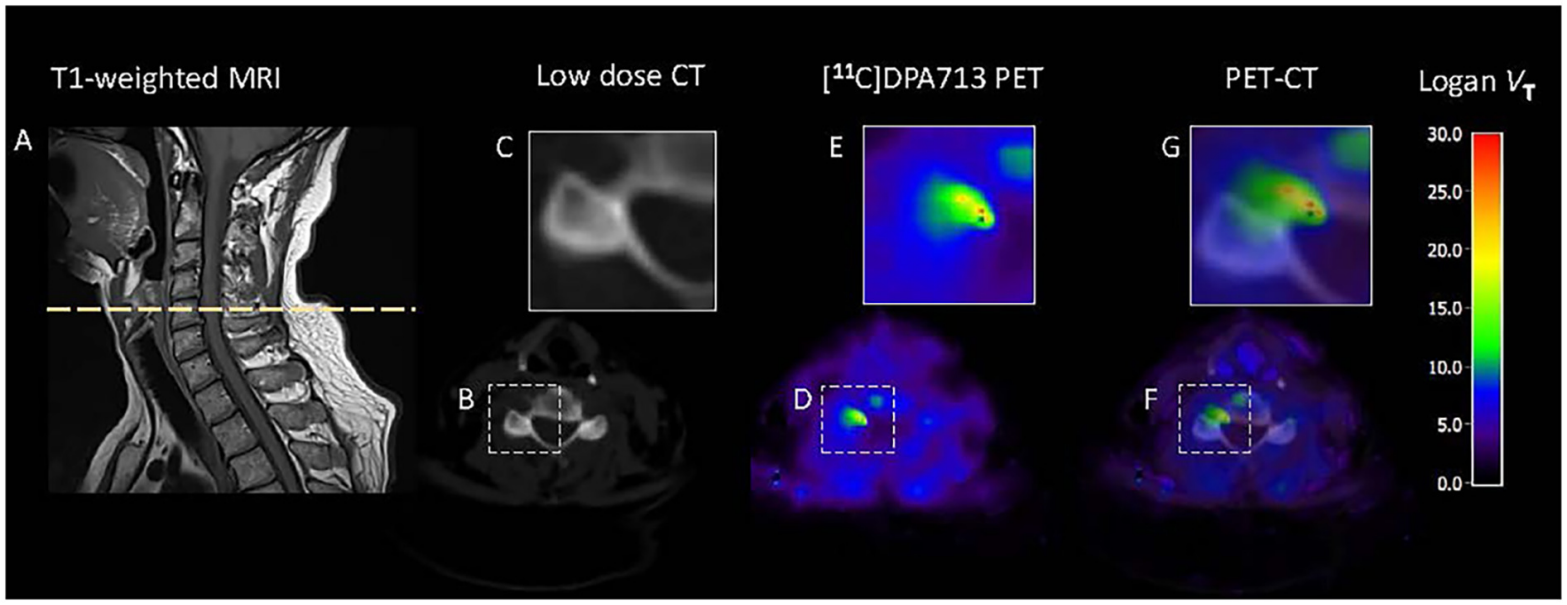
Graphical summary. Neuroinflammation in a patient with painful cervical radiculopathy C6. Patient was a mixed-affinity binder for TSPO polymorphism. To quantify [^11^C]DPA713 binding at the neuroforamina and spinal cord, a single tissue compartment (1T2k) model with image derived input function was used. For visualization purposes, volume of distribution (*V*_T_) images were generated using Logan plot analysis, with a time threshold of t* = 30 min. **(A)** T1-weighted MRI. The dotted line indicates the cross-sectional area used for the CT and PET analysis. **(B)** Low-dose CT cross-sectional image, with **(C)** a zoomed view at the neuroforamina. **(D)** A parametric cross-sectional image of [^11^C]DPA713 binding, with **(E)** a zoomed view at the neuroforamina. Higher volume of distribution (*V*_T_) indicates more tracer binding, suggesting higher levels of neuroinflammation. **(F)** CT and PET images merged with **(G)** a zoomed view at the affected neuroforamina.

These results indicate that a 1T2k model with a metabolite-corrected image-derived input function can be used to describe the kinetics of [^11^C]DPA713 in the spinal cord and neuroforamina in humans (Figure 5). 1T2k *V*_T_ or Logan *V*_T_ can be used as binding metrics, while TBR-PP is the recommended choice among simplified models. PET may offer added value in the clinic by revealing where neuroinflammation is present, potentially aiding in understanding the spatial distribution and dynamics of inflammation in chronic radiculopathy (49). Although dynamic PET is not yet suitable for routine clinical use, it holds promise for advancing our understanding of the complex pathophysiology of painful cervical radiculopathy and the effects of treatments. However, to interpret treatment effects reliably, it is essential to use a metabolite-corrected input function, as therapeutic interventions may alter both the blood signal and the proportion of radiolabelled metabolites. Without this correction, changes in tracer kinetics could be misattributed to treatment effects rather than underlying pharmacokinetic alterations.

## Data Availability

The raw data supporting the conclusions of this article will be made available by the authors, without undue reservation.
