# Supplementary material for: Quantification of neuroinflammation in spinal cord and neuroforamina of patients with painful cervical radiculopathy using [11C]DPA713 PET/CT
Source: Front Nucl Med. 2025 Aug 21;5:1569991. doi: 10.3389/fnume.2025.1569991 (PMC12408627; doi:10.3389/fnume.2025.1569991)
Supplement: Supplementary file 1 [file Table1.docx]

**SUPPLEMENTARY FIGURE 1**

**Supplementary 1.** Visual presentation of an outlier for the 1T2k model due to patient motion at the neuroforamina. **A:** Represents the fits for the peak only; **B:** Represents the fit over the whole scan session. AIC for 1t2k: -80.8, AIC for 2t3k: -93.3 and AIC for 2t4k: -91.2.

**SUPPLEMENTARY FIGURE 2**


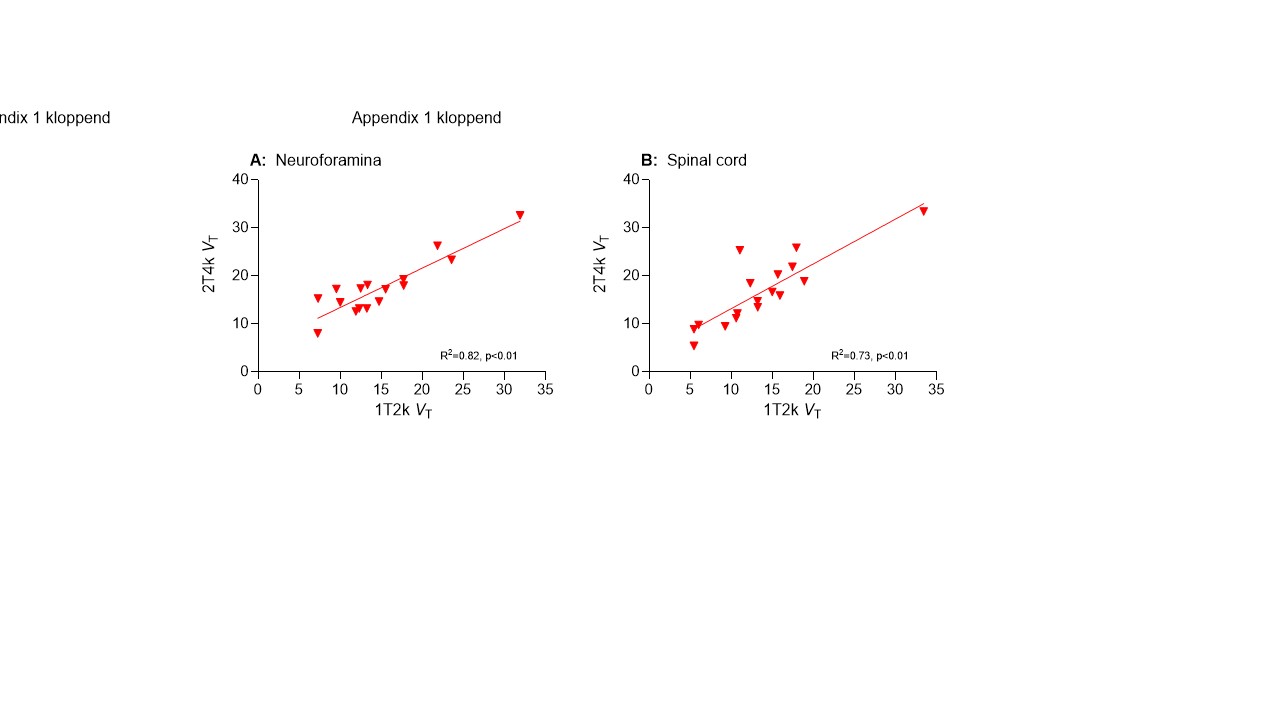


**Supplementary 2**. Correlation between 1T2k *V*_T_ and 2T4k *V*_T_. Abbreviations: *V*_T_: Volume of distribution; 1T2k: single-tissue compartmental model; 2T4k: two-tissue reversible compartmental model; R^2^: explained variance.

**SUPPLEMENTARY FIGURE 3**


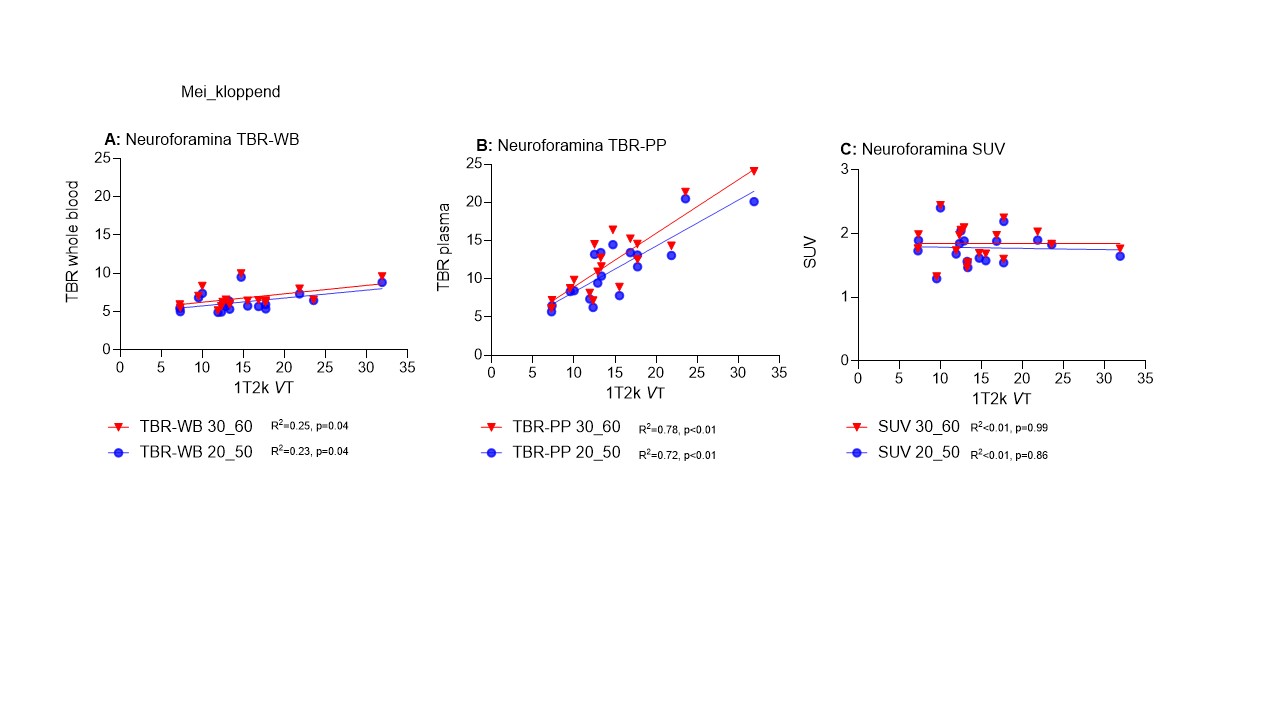


**Supplementary 3**. Association between simplified outcome measures and 1T2k *V_T_* for affected neuroforamina in patients with cervical radiculopathy. Abbreviations: *V*_T_: Volume of distribution; 1T2k: single-tissue compartmental model; SUV: standardized uptake value; TBR-WB: target-to-whole blood ratio; TBR-PP: target-to-metabolite corrected plasma ratio.

**SUPPLEMENTARY TABLE 4**

**Supplementary 4A:** 1T2k Volume of distribution with and without metabolite correction

| **Patient** | **Time moment** | **1T2k_WB *V*_T_** | **1T2k *V*_T_** | **Metabolites, %** |
| --- | --- | --- | --- | --- |
| 01 | Test | 12.21 | 21.84 | T1: 18.12; T2: 28.13; T3: 39.66; T4: 38.81; T5: 44.35; T6: 27.74; T7: 26.39. |
|  | Retest | 11.02 | 12.32 | T1: 9.39; T2: 12.83; T3: 14.48; T4: 21.45; T5: 10.19; T6: 16.00; T7: 14.55 |
| 02 | Test | 6.21 | 23.56 | T1: 55.05; T2: 62.08; T3: 67.10; T4: 72.79; T5: 70.97; T6: 74; T7: 71.01 |
|  | Retest | 8.84 | 17.71 | T1: 26.78; T2: 38.77; T3: 38.03; T4: 40.35; T5: 46.97; T6: 44.30; T7: 58.25 |

**Abbreviations:** *V*_T_: Volume of distribution; 1T2k: single-tissue compartmental model; 1T2k_WB *V*_T_: metabolite uncorrected; T1: 5 minutes post injection; T2: 10 minutes post injection; T3: 15 minutes post injection; T4 30 minutes post injection; T5 : 35 minutes post injection ; T6 : 40 minutes post injection ; T7 : 59 minutes post injection.

**Supplementary 4B:** 1T2k Volume of distribution with and without metabolite correction

|  | Test  mean (SD) | Retest  mean (SD) | Mean difference (SD) | r | R^2^, p-value | ICC_2.1_ (95%CI), p-value | SEM | SDD | LoA (LL, UL) |
| --- | --- | --- | --- | --- | --- | --- | --- | --- | --- |
| Neuroforamina | | | | | | | | | |
| 1T2k *V*_T target tissue_ | 16.64 (5.02) | 13.63 (3.91) | 3.01 (4.31) | 0.56 | 0.32, 0.25 | 0.64 (-0.55, 0.95), 0.10 | 2.02 | 5.60 | -11.0, 5.4 |
| 1T2k_WB *V*_T target tissue_ | 11.28 (4.91) | 13.89 (6.77) | 2.60 (3.42) | 0.88 | 0.77, 0.02 | 0.88 (0.22, 0.98), 0.01 | 2.02 | 5.60 | -4.1, 9.3 |
| Spinal cord | | | | | | | | | |
| 1T2k *V*_T target tissue_ | 16.83 (8.87) | 11.86 (3.71) | 4.96 (7.32) | 0.59 | 0.35, 0.22 | 0.53 (-0.85, 0.93), 0.17 | 4.31 | 11.96 | -19.0, 9.4 |
| 1T2k_WB *V*_T target tissue_ | 10.21 (6.65) | 9.57 (3.14) | 0.64 (4.93) | 0.71 | 0.50, 0.11 | 0.74 (-1.45, 0.97), 0.10 | 2.49 | 6.91 | -10, 9.0 |

Intraclass coefficient between 1T2k *V*_T_ with and without metabolite correction was fair to good (ICC_2.1:_ 0.75 (95% CI: 0.51, 0.87), p<0.001.

**Abbreviations:** *V*_T_: Volume of distribution; 1T2k: single-tissue compartmental model; 1T2k_WB *V*_T_: metabolite uncorrected; ICC: intraclass coefficient; SEM: standard error of the mean; SDD: smallest detectable difference; LOA: limits of agreement; LL: lower limit; UL: upper limit; SD: standard deviation.

**Supplementary 4C:** Absolute agreement test-retest metabolite fractions

|  | ICC_3.1_ (95%CI), p-value |
| --- | --- |
| Patient 1 | 0.11 (-0.19, 0.64), 0.30 |
| Patient 2 | 0.22 (-0.06, 0.72), 0.03 |
| Patient 3 | 0.24 (-3.77, 0.87), 0.38 |
| Patient 4 | 0.12 (-0.05, 0.57), 0.08 |
| Patient 5 | 0.24 (-0.05, 0.74), 0.02 |
| Patient 6 | 0.11 (-0.27, 0.68), 0.34 |
| Combined (n=6 patients) | 0.70 (-0.20, 0.90), <0.01 |
